# The Impact of Sedentary Lifestyle, High-fat Diet, Tobacco Smoke, and Alcohol Intake on the Hematopoietic Stem Cell Niches

**DOI:** 10.1097/HS9.0000000000000615

**Published:** 2021-07-19

**Authors:** Katja Kaastrup, Kirsten Grønbæk

**Affiliations:** 1 Department of Hematology, Rigshospitalet, Copenhagen, Denmark; 2 Biotech Research and Innovation Centre (BRIC), University of Copenhagen, Denmark; 3 Novo Nordisk Foundation Center for Stem Cell Biology (DanStem), Faculty of Health Sciences, University of Copenhagen, Denmark

## Abstract

Hematopoietic stem and progenitor cells maintain hematopoiesis throughout life by generating all major blood cell lineages through the process of self-renewal and differentiation. In adult mammals, hematopoietic stem cells (HSCs) primarily reside in the bone marrow (BM) at special microenvironments called “niches.” Niches are thought to extrinsically orchestrate the HSC fate including their quiescence and proliferation. Insight into the HSC niches mainly comes from studies in mice using surface marker identification and imaging to visualize HSC localization and association with niche cells. The advantage of mouse models is the possibility to study the 3-dimensional BM architecture and cell interactions in an intact traceable system. However, this may not be directly translational to human BM. Sedentary lifestyle, unhealthy diet, excessive alcohol intake, and smoking are all known risk factors for various diseases including hematological disorders and cancer, but how do lifestyle factors impact hematopoiesis and the associated niches? Here, we review current knowledge about the HSC niches and how unhealthy lifestyle may affect it. In addition, we summarize epidemiological data concerning the influence of lifestyle factors on hematological disorders and malignancies.

## Modifiable lifestyle factors and the risk of hematological malignancies

Lifestyle-related diseases such as cardiovascular diseases, diabetes, chronic respiratory diseases, and cancer may all be preceded by exposure to one or more modifiable lifestyle factors such as unhealthy diet, physical inactivity, excessive alcohol intake, and smoking.

The role of lifestyle factors in the development of hematopoietic cancer is still unclear. However, epidemiological studies show that obesity 2 years before diagnosis are associated with acute myeloid leukemia (AML) for both males and females, whereas obesity is only associated with myelodysplastic syndrome (MDS) in females.^1^ Furthermore, obesity in adolescence has been associated with increased risk of developing myeloproliferative neoplasms.^2^ The data regarding the influence of alcohol on the development of MDS are conflicting. Some studies report that alcohol is not a significant contributor to MDS,^3,4^ whereas a case-control study in Japanese men report a direct correlation between the amount of alcohol consumed per week and the risk of MDS.^5^ Large epidemiological studies incorporating biomarkers of exposure (eg, gamma-glutamyl transferase) are needed to better understand the contribution of alcohol intake to the development of MDS. Interestingly, heavy alcohol intake has been associated with a lower risk of non-Hodgkin lymphomas.^6,7^ Smoking on the other hand significantly increases the risk of hematologic malignancies,^6,8,9^ including an increase in the relative risk of developing MDS and AML by up to 40% in active smokers, and 25% in former smokers.^10^ In addition, smoking is significantly associated with myeloid clonal hematopoiesis,^11^ which is a risk factor for hematological cancer.

Specific alterations of the hematopoietic stem cell (HSC) niches may predispose to hematological cancer by facilitating mutant HSC survival and expansion.^12^ Exposure to modifiable lifestyle factors may introduce toxic or unhealthy substances to the circulation and/or increase body weight which potentially lead to changes in the HSC niche. However, most of the current knowledge comes from studies in mice, and the exact mechanisms by which lifestyle factors affect the niche in human malignant hematopoiesis is a challenging issue for further investigation.

## The hematopoietic niches

The functionality of HSCs depends on the balance between quiescence and activation. Reduced ability of HSCs to escape quiescence and initiate proliferation results in insufficient blood cell production. On the other hand, too many HSCs leaving quiescence or failing to return to quiescence after activation may exhaust the HSC pool resulting in bone marrow (BM) failure. Thus, HSC quiescence is crucial for sustaining the HSC pool and protects the HSCs by minimizing replication-associated mutations in their genome.^13^ The concept of the HSC niche was proposed to define the cellular interactions and molecular pathways that underlie the regulation of quiescence or activation of individual HSCs by their BM microenvironment.

## The BM architecture

The BM resides within the cavities of axial and long bones, which consist of a delicate combination of mineralized tissue, BM, endosteum, periosteum, blood vessels, and nerves. The inner surfaces of the bone cavities are covered by a layer of flat-bone lining cells in a structure known as the endosteum.^14^ Located in the endosteum are the osteoblasts and osteoclasts.^15^

In long bones, arteries pass through bone canals entering the BM cavity and branch into a multitude of arterioles, capillaries, and sinusoids creating a vascularized BM region.^14,16^ The BM microvasculature supplies oxygen and nutrients and removes metabolic waste from the extensively productive BM. Furthermore, mature blood cells leave the BM to the systemic circulation through the sinusoids.

There are divergent data on whether the HSCs reside in endosteal or perivascular niches, and whether the perivascular niche is sinusoidal or arteriolar. Several studies in mice report a perivascular localization of HSCs.^17,18^ Studies of human HSC niches are limited. One study using human BM biopsies reports that HSCs primarily localize to endosteal regions while hematopoietic stem and progenitor cells (HSPCs) reside in both vascular and endosteal regions.^19^ However, another study reports a perivascular localization of HSCs.^20^ The inconsistencies in HSC localization may indicate a dynamic transition of HSCs to multiple niches and/or reflect a different proliferative status or reconstitution potential of the individual HSCs.

In support of this, human HSCs localizing close to bone (trabecular area) in xenograft mouse models have superior repopulating and self-renewal capacity and are molecular distinct from those localizing in the central portion of BM.^19^ The HSCs localizing close to trabeculae express genes involved in HSC self-renewal and BM niche retention, whereas genes involved in proliferation and survival are all downregulated.^19^ This suggests a quiescent state of HSCs locating near the endosteum. Similarly, it has been suggested that quiescent mouse HSCs preferentially localize to arterioles, whereas cycling HSCs localize more closely to sinusoids.^21^ Recent studies in mice further support the hypothesis of a dynamic niche as they show that HSCs possess some motility, especially upon activation, and can be found at both perivascular and endosteal regions.^22,23^

## Cellular and molecular niche factors

There are multiple cells that regulate murine HSC physiology; osteoblasts, endothelial cells, mesenchymal stromal cells (MSCs), macrophages, megakaryocytes, hematopoietic elements, osteoclasts, and cells of the nervous system.^24^ Any individual cell within the 3-dimensional environment may make direct contact with multiple cell types and receive biochemical information from others through surface-expressed, secreted or otherwise transferred signaling molecules.^24^ Such signaling molecules include, among many others, the well-studied chemokine C-X-C motif ligand 12 (CXCL12), stem cell factor (SCF), and Notch ligands. As in mouse, the human BM microenvironment is complex and consists of bone and its lining cells osteoblasts and osteoclasts, endothelial cells from various vasculature, MSCs, and resident mature blood cells.^24,25^

BM MSCs are multipotent mesenchymal precursor cells that have the ability to differentiate into osteoblasts, adipocytes, and chondrocytes.^26^ In mouse BM, MSCs are strikingly abundant and form a dense network via extension of numerous, elongated, thin processes that run along extracellular matrix fibers. As a consequence, the entire BM space are within <7 μm of the nearest MSC surface.^27^ The MSC population is likely heterogenous with multiple subpopulations with distinct molecular profiles and locations.^21,28–30^ MSCs are known to have high expression of CXCL12 and/or SCF.^28,31^ CXCL12 is an important niche factor which promotes HSC maintenance and lymphopoiesis.^32^ Furthermore, CXCL12 serves as a potent chemoattractant for CXC chemokine receptor 4-positive hematopoietic cells and therefore is critical for BM homing and HSC retention.^33,34^ SCF preserves the viability of HSPCs^35^ and facilitates their proliferation and differentiation.^36^

Recently, 2 previously unknown MSC populations were identified in mouse BM. Interestingly, the 2 populations express adipocyte and osteo-lineage genes differentially and are termed “Adipo-CXCL-12 abundant (CAR)” and “Osteo-CAR” cells, respectively. The Adipo-CAR cells are predominantly found in sinusoidal areas, whereas Osteo-CAR cells are in arteriolar or nonvascular areas, suggesting that the 2 populations occupy distinct niches.^37^ Similarly, a study of human xenograft mouse models shows that osteoblasts in trabecula areas have increased expression of Notch ligands (Jagged-1, Jagged-2, and Delta-like 4) compared to osteoblasts in long bone area.^19^ Furthermore, CXCL12 from osteoblasts is required for the maintenance of early lymphoid progenitors in mice but not for HSCs or myeloid progenitors, suggesting a preferential endosteal niche for the lymphoid lineages in mice.^32^

Endothelial cells that line the BM blood vessels also contribute to the mouse HSC niche through expression of the Notch ligand Jagged-1 and SCF.^38,39^ Coculture studies of mouse-derived endothelial cells and HSCs have shown that expression of Notch ligands by endothelial cells promotes proliferation and prevents exhaustion of HSCs.^40^ Furthermore, expression of Jagged-1 by endothelial cells regulates self-renewal of HSCs in vivo, as evidenced by a profound decrease in hematopoiesis and premature exhaustion of HSCs upon conditional deletion of Jagged-1 in endothelial cells.^39^

## BM adipocytes: a HSC niche component?

The adult human BM consists of 50 to 70% adipose tissue. There is emerging evidence that BM adipocytes (BMAs) exert functions beyond mere filling of BM empty spaces as previously anticipated, but do they regulate hematopoiesis?

BMAs arise from differentiation of BM MSCs. A recent study in mice revealed that BMAs are in close proximity to both sinusoidal vessels, cells of the myeloid and granulocyte lineage, and osteoblasts.^41^ Although the human BMA morphology resembles that of white adipocytes with a large lipid droplet, they comprise a heterogenous population with distinct lipid profile and metabolism,^42^ gene expression,^43^ functional responses,^44^ and localization,^45^ and they have been assigned the color yellow.^46^

The existence of 2 types of BM adipose tissue (BMAT) in mice has been suggested: regulated marrow adipose tissue (rMAT) and constitutive marrow adipose tissue (cMAT). rMAT presents as single adipocytes located at sites of active hematopoiesis, whereas cMAT contains larger adipocytes and are found in regions with a low number of hematopoietic cells. cMAT develops earlier and remains preserved on systemic challenges.^47^

Adipocytes are highly active secretory cells and release a large variety of factors, including SCF, to the local BM environment. The BMAs have been proposed as being a niche component during emergency hematopoiesis, that promotes hematopoietic regeneration after irradiation. After exposure to irradiation or chemotherapy, endothelial cells and subsets of MSCs are depleted in mice, while adipocytes become abundant. Indeed, depletion of SCF from BMAs has little effect on SCF protein levels in mice with nonirradiated BM, but substantially reduces SCF levels in irradiated BM.^48^

Conversely, BMAs have also been implicated as predominantly negative regulators of hematopoisis.^49^ HSPCs are 2- to 3-fold reduced in adipocyte-rich BM of the tail vertebrae compared to nonadipocytic BM from the thoracic vertebrae in mice.^49^ Moreover, coculture studies using mouse BM–derived cells have shown that BMAs inhibit regeneration of hematopoiesis by inducing apoptosis in HSPCs and by secreting transforming growth factor β1 (TGF-β1), which have been implicated in the regulation of proliferation, quiescence, and differentiation of HSCs.^50,51^ Furthermore, adipocytes are known to secrete dipeptidyl peptidase-4,^52^ which cleaves important niche factors such as CXCL12.^53^ In agreement with the in vitro experiments, adipocyte progenitor cells and preadipocytes transplanted into the tibia of mice lead to a significant reduction of hematopoietic progenitor cells.^54^

Currently, little is known about the physiology of human BMAT. A recent study show that healthy elderly individuals (65-92 years) have significantly more HSPCs located immediately adjacent to adipocytes compared with middle-aged healthy individuals (50-64 years).^55^ Furthermore, elderly have an increase in total number of myeloid cells, a decrease in lymphoid cells, and a higher density of maturing myeloid cells surrounding adipocytes compared with middle-aged healthy individuals, which may influence myeloid skewing and the risk of myeloid malignancies; all together suggesting a negative regulation by BMAs on human hematopoiesis.

## Lifestyle factors and their influence on HSC niches

### High-fat diet are associated with a loss of stemness in HSCs and MSCs

For many years, it has been known that diet affect hematopoiesis examples being anemia in iron^56^ and folate deficiency.^57^ In 2016, almost 40% of the globe's adults were overweight^58^ and the incidence is increasing. The increase in prevalence of obesity is often attributed to increased fat intake and decreased physical activity.^59^

Low-grade inflammation is a hallmark of obesity demonstrated by increased circulating levels of proinflammatory cytokines such as interleukin (IL)-6, IL-1β, and tumor necrosis factor alpha (TNF-α).^60^ However, the BMAs from obese mice^61^ and BM MSCs from obese humans^60^ do not exhibit a proinflammatory phenotype. Thus, more studies are needed to elucidate whether systemic inflammation during obesity impacts inflammation in the BM and the HSC niches.

Several studies in mice report that high-fat diet (HFD) and/or diet-induced obesity cause loss of HSC quiescence and induce differentiation, resulting in a shift from self-renewing HSCs toward mature progenitors.^62–64^ HFD has been reported to create a myeloid bias in mice and to decrease expression of CXCL12 and Jagged-1.^64^ Similarly, obese humans have higher numbers of granulocytes and monocytes in peripheral blood consistent with increased myelopoiesis.^65^ Whether the decrease in CXCL12 and Jagged-1 can explain the loss of stemness of HSCs during obesity is a subject for further research.

**Figure 1.**
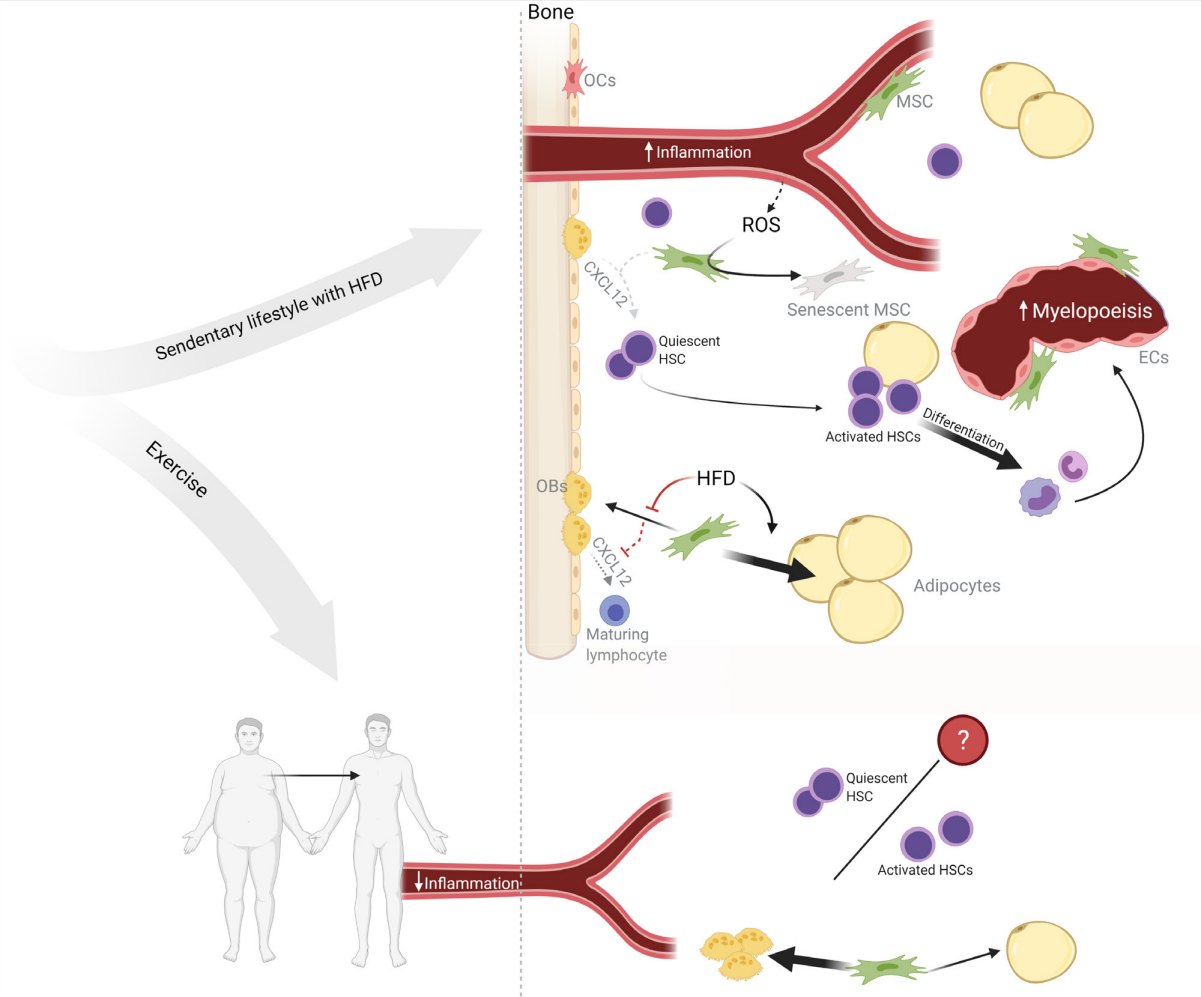
**Hypothetical working model for obesity-induced niche changes in human BM.** HFD decreases CXCL12 expression in the BM, induces HSC activation and differentiation resulting in a shift from self-renewing HSCs toward mature progenitors with a myeloid bias. Obesity increases circulating levels of proinflammatory cytokines and ROS. MSCs of obese BM are senescent and exhausted possibly mediated by the increased ROS levels. Obesity/HFD suppress osteogenic and induce adipogenic differentiation of MSCs leading to an expansion of BMAT. Exercise counteracts the systemic effect of obesity by improving body composition and decreasing the systemic inflammation. Furthermore, exercise may improve the obesity-induced niche changes by decreasing the amount of BMAT and increasing osteoblast differentiation. Furthermore, exercise may balance the quiescence versus active state of HSCs. BM = bone marrow; BMAT = bone marrow adipose tissue; CXCL12 = C-X-C motif ligand 12; ECs = endothelial cells; HFD = high-fat diet; HSC = hematopoietic stem cell; MSC = mesenchymal stromal cell; OBs = osteoblasts; OCs = osteoclasts; ROS = reactive oxygen species. Created with BioRender.

Studies on mice MSCs suggest that obesity skews differentiation of MSCs toward adipocytes at the expense of osteoblasts.^54,64^ As described previously, CXCL12 from osteoblasts are important for the maintenance of lymphoid progenitors, and adipocyte over osteoblast differentiation could potentially have a negative impact on production of lymphoid cells. Moreover, obesity has been shown to increase reactive oxygen species (ROS) production which is associated with BM MSCs senescence and stem cell exhaustion^60^ (Figure 1).

Similarly, obesity in humans has been associated an enrichment of adipocyte progenitors in the BM.^60^ While the direct impact of increased BMAT on the HSC niches is yet unknown, increased BMAT would bring many more hematopoietic and niche cells into closer contact with BMAs. Increased BMAT during aging has been associated with reduced hematopoiesis.^55^ In addition, transfer of fatty acids from increased lipolysis in BMAT has been reported to support the proliferation and survival of AML blasts from patients.^66^ This is an interesting finding since obesity^1,67,68^ and type 2 diabetes^69^ are known risk factors for hematological malignancies. Of note, BMAs also have an important role in emergency hematopoiesis as described earlier. Prolonged dietary-induced weight loss is associated with a decrease in BMAT in humans with the largest effect observed in those with higher baseline BMAT.^70^ However, the reported effect may be transient as the amount of BMAT increases after the diet-intervention ended.

Paradoxically, the amount of BMAT also increases in cases on chronic malnutrition/starvation as seen in anorexia nervosa (AN).^71–74^ This is attributed an increased adipocyte over osteoblast differentiation^72^ as with the HFD BM. Low blood cell counts are frequently observed in patients with AN.^75,76^ These data suggest that metabolic stress, either through starvation or obesity, results in similar BM alterations with increased adipocyte differentiation causing an increase in BMAT. However, the effect on hematopoiesis seems to vary as HFD has been associated with myelopoiesis and starvation is generally associated with low blood cell counts. These differences may arise from deficiencies in important nutrients, vitamins, and minerals^77^ with impact on hematopoiesis, which are frequently seen in patients with AN. Indeed, protein malnutrition suppresses cell cycle progression in HSPCs,^78^ leads to anemia and leukopenia, decreases the production of CXCL12 and SCF from endothelial cells,^79^ and decreases trabecular bone^80^ in mice.

A healthy body weight results from balancing adequate diet and physical activity. Worldwide physical inactivity is increasing, and in 2016, 28% of adults aged 18 and over did not meet the global recommendations of physical activity.^81^

### Exercise may reverse the obesity-induced rise in BMAs and increase numbers of HSPCs in BM

Exercise has numerous benefits on the human physiology. Consistent exercise counteracts the systemic effect of obesity by improving body composition^82^ and decreasing low-grade inflammation.^83^

Some studies have shown that exercised humans^84,85^ and mice^86,87^ have increased amounts of HSPCs in BM and peripheral blood. Furthermore, exercise induces a short and rapid mobilization of HSPCs and endothelial cells to the circulation. Divergent data exist on whether exercise increases HSC quiescence^88^ or activation^86^ in mice, but in general, there is a consensus that exercise does not exhaust or impair HSCs.

MSCs from exercised-trained mice have decreased adipocyte and increased osteoblast differentiation potential.^87^ However, 1 study reported no difference in BM osteoblast numbers in running versus sedentary mice.^88^ Mechanical load or strain to BM MSCs in vitro largely downregulate adipogenesis and promote differentiation of osteoblasts.^89,90^ Interestingly, regular exercise decreases the amount of BMAT both in humans^91,92^ and mice.^87,93^

Overall, the effect of exercise on the BM is still not well described, but current data suggest that exercise may counteract the effect of obesity-induced changes in the HSC niche (Figure 1). The effects of exercise on the individual cell populations within both the endosteal and perivascular niches, and on hematopoiesis in general, is an interesting subject for further investigation.

In addition to the increased prevalence of obesity, possibly attributed a HFD and lack of exercise, the total volume of alcohol consumed has increased by 70% from 1990 to 2017.^94^ Heavy alcohol intake may also induce systemic inflammation and increase BMAT as seen with obesity.

### Alcohol metabolites are potentially harmful to the BM

Excessive alcohol consumption is a major public health concern, and a risk factor for increased mortality from cancer, hepatic disorders, diabetes, infections, and BM suppression.^95,96^ Ingested alcohol is rapidly absorbed through the gastrointestinal tract into the bloodstream and distributed throughout the water in the body, so that most tissues are exposed to the same concentration of alcohol as the blood.^97^ More than 90% of alcohol is metabolized by the liver. Metabolic pathways of alcohol involve the oxidation of alcohol to acetaldehyde by the enzymatic activity of alcohol dehydrogenase. Acetaldehyde is a highly reactive and toxic substance, and is in healthy people rapidly oxidized to acetate by aldehyde dehydrogenase.^97^ The reduction of nicotinamide adenine dinucleotide (NAD) is required in both reactions.^98^ Several isozymes of aldehyde dehydrogenase exist and 1 is absent in about 40% in the Japanese population.^99^

Overall, alcohol metabolism results in production of acetaldehyde and acetate, enhanced activity of the respiratory chain due to NADH formation, and increased iron uptake, which all promote the generation of ROS.^100^

### Excessive alcohol consumption increases oxidative stress in the BM causing adipocyte over osteoblast differentiation and deterioration of the bone microarchitecture

A recent study demonstrated for the first time that chronic voluntary alcohol drinking in rhesus macaque monkeys leads to long-term impairment of HSPC function, and alterations in the BM microenvironment that consist after 1 month of abstinence.^101^ Purified HSPCs from alcohol-drinking monkeys produce significantly less granulocyte-monocyte and erythroid colonies in vitro compared with cells from control animals.^101^ These data are consistent with the clinical observation that excessive alcohol consumption is associated with granulocytopenia, thrombocytopenia, and anemia. The liver is the site of production of thrombopoietin.^102^ Accordingly, alcohol-induced liver damage significantly contributes to perturbed thrombopoiesis.

Chronic excessive alcohol consumption is also considered a high-risk factor for loss of bone mineral density, impairment of bone remodeling, and deterioration of bone tissue,^103,104^ and is a cause of osteoporosis.^105^ Multiple studies report that alcohol have an inhibitory effect on osteogenic differentiation of human BM MSCs in vitro through stimulation of oxidative stress that suppresses Wnt signaling.^106–109^ Furthermore, ethanol treatment induces premature senescence in cultured human BM MSCs with decreased cell proliferation and cell cycle arrest in a dose-dependent manner possibly by increasing ROS levels.^106^

Importantly, ROS levels are significantly higher in serum of patients with alcohol dependence compared with healthy controls^110^ indicating that other organs and tissues than the liver are exposed to high levels of ROS during ethanol metabolism. Furthermore, ethanol can freely diffuse into cells and induce intracellular oxidative stress in cultured human osteoblasts.^111^ The effects of alcohol on cultured cells were partly confirmed in an in vivo study in mice, where high doses of ethanol downregulate osteogenic differentiation of BM MCSs, whereas low doses improve osteogenic differentiation and elevate bone formation.^112^ This suggests that multiple mechanisms may contribute to alcohol-induced bone loss, and that the effect may depend on the amount of alcohol consumed. In addition, ethanol significantly increases the number of BMAs and expression of genes important for adipocyte differentiation in cultured human BM MSCs^113^ and in mice.^112^ Thus, ethanol induces an adipocyte over osteoblast differentiation of MSCs, which is well in line with the clinical observation of increased risk of osteoporosis with chronic alcohol consumption.

In patients with excessive alcohol consumption, acetaldehyde-derived epitopes have been found in peripheral blood erythrocytes and their precursors in BM,^114^ suggesting a direct role in hematotoxicity, which is consistent with studies on cultured murine and human HSPCs^115^ and osteoblast cell lines.^116^ Since HSCs mainly reside in a quiescent state, and are long-lived, they are continuously exposed to potential genotoxic agents such as acetaldehyde. A more recent study in mice reports that the primary protection against acetaldehyde is provided by aldehyde dehydrogenase–mediated detoxification. However, when this is lost or saturated, acetaldehyde induces DNA damage in HSCs, and HSCs mutated by aldehydes are functionally compromised and display myeloid bias.^117^

Bone microarchitecture is not only an important determinant of bone strength but also an important structural component of the HSC niches, as different cell types have unique molecular functions based on their location to, among others, trabecular bone areas. Reduction in trabecular bone volume has been described in multiple animal models following ethanol diet.^118–122^

**Figure 2.**
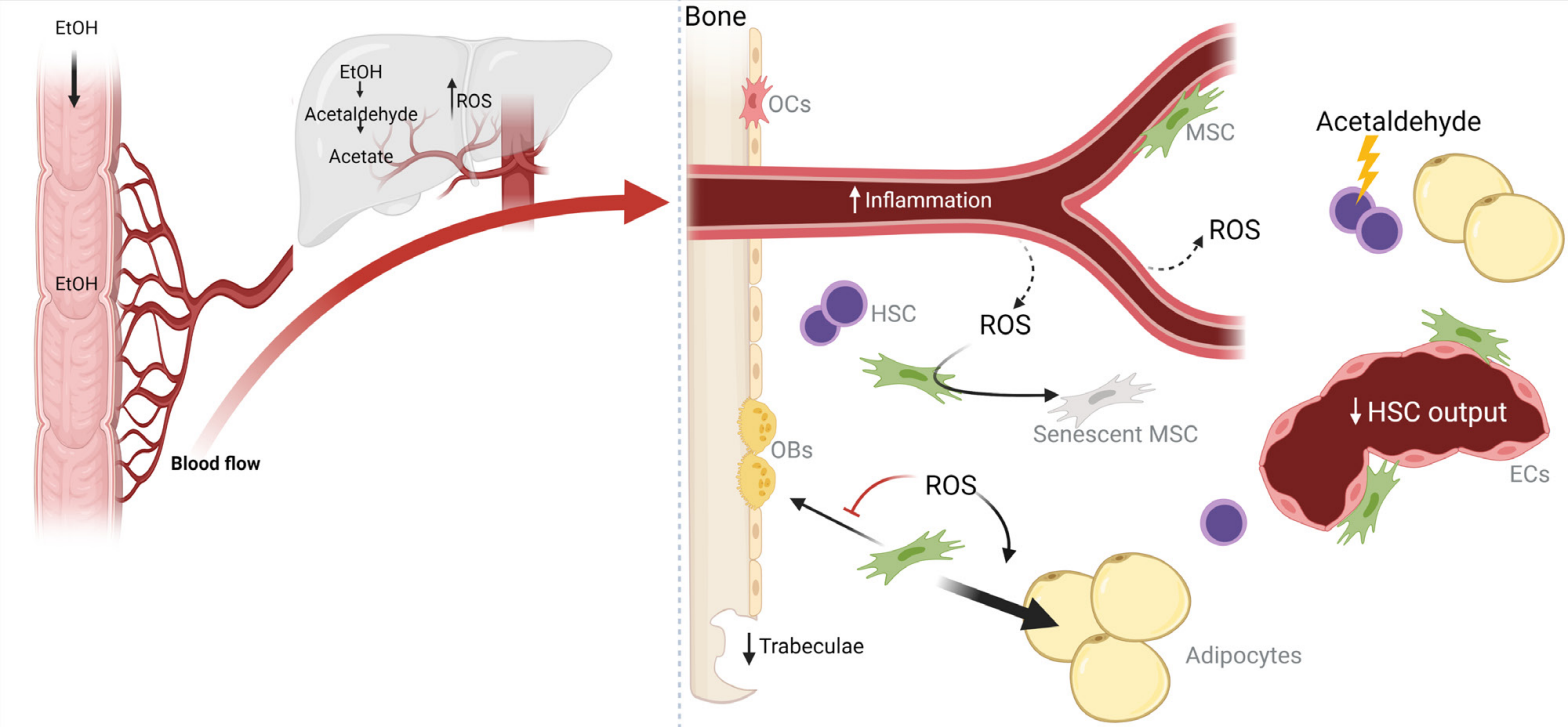
**Hypothetical working model for ethanol-induced niche changes in human BM.** Ingested alcohol is rapidly absorbed through the gastrointestinal tract into the bloodstream. Ethanol is metabolized mainly in the liver to acetaldehyde and acetate, which generates high levels of ROS. Chronic excessive alcohol consumption is associated with pancytopenia and increased circulatory levels of proinflammatory cytokines. However, the inflammatory status of the HSC niche during excessive alcohol consumption remains unknown. The increased ROS levels induce MSC senescence and reduced proliferation. Furthermore, ROS inhibit osteogenic and promote adipogenic differentiation of MSCs. In addition, ethanol diet reduces trabecular bone volume. Acetaldehyde is directly hematotoxic and induces DNA damage in HSCs. Normally, acetaldehyde is rapidly converted to acetate by aldehyde dehydrogenase, but the enzymes may be lost or saturated leading to increased acetaldehyde. BM = bone marrow; ECs = endothelial cells; EtOH = ethanol; HSC = hematopoietic stem cell; MSC = mesenchymal stromal cell; OBs = osteoblasts; OCs = osteoclasts; ROS = reactive oxygen species. Created with BioRender.

There is a lack of data addressing alcohol-induced changes in the BM microenvironment and microarchitecture in humans. However, a few studies on alcohol abuse report reduced trabecular bone volume in humans and confirm the low osteoblast activity.^123,124^ Studies on the effect of alcohol consumption in humans are challenged by confounders and comorbidity factors among patients such as variations in consumption, alcohol percentage, age, sex, ethnicity, impaired nutritional status, behavioral differences, and the presence of comorbidities such as liver insufficiency and other organ damage. However, the current data suggest potential alcohol-induced changes to the HSC niches such as decreased osteoblast numbers, increased numbers of BMAs, and remodeling of the bone microarchitecture (Figure 2). These changes could theoretically lead to changes in the HSCs resulting in impaired hematopoiesis consistent with the clinical presentation of patients with excessive alcohol consumption. However, more studies are needed to evaluate the mechanistic effect of ethanol on the HSC niches and the resulting consequences on human hematopoiesis.

### Prolonged alcohol consumption promotes the development of inflammation

Prolonged alcohol consumption is associated with increased blood levels of proinflammatory cytokines, such as IL-6,^125,126^ IL-8,^125,127^ Regulated upon Activation, Normal T cell Expressed and Secreted,^128^ and TNF-α.^129^ Inflammation triggers a protective response involving blood and immune cells and leads to drastic alterations in hematopoietic output to compensate for the increased demand.^130^

The role of the HSC niches during inflammation is still uncertain. MSCs express various cytokine/chemokine receptors enabling them to sense any local or systemic inflammation. On activation of cytokine receptors, the MSCs are able to modify the cytokine and chemokine profile in the environment through production of additional factors.^131^ Thus, it is likely that MSCs in both the endosteal and perivascular niche activate inflammatory programs during chronic alcohol consumption, which may directly affect the maturation of neighboring HSPCs. However, this remains hypothetical and future studies are needed to address whether the cellular components of the HSC niches adapt their hematopoiesis-supporting functions according to the inflammatory status of the BM, and whether such changes are persistent or reversible during an abstinent period.

A sustained inflammatory response may result in HSC exhaustion, accumulation of genetic alterations, and BM failure, all of which may promote the development of hematopoietic malignancies.^132^ Considering the alcohol-induced increased ROS levels, inflammation, and niche remodeling, it is surprising that heavy alcohol drinking has been associated with a lower risk of non-Hodgkin lymphomas.^6,7^

Systemic inflammation may also be caused by other external stimuli, such as cigarette smoke.

### Cigarette smoking reduces the number of BM MSCs and endothelial progenitor cells

Inhaled tobacco smoke reaches the airways, and nicotine and other compounds are absorbed into the bloodstream.^133^ Multiple studies report that smoking induces leukocytosis and increased blood cell counts.^134–136^ Studies exposing various animal models to cigarette smoke report inhibition of the number and function of MSCs.^137–139^ Similarly, cigarette smoke extract injected intraperitoneally in mice depletes BM endothelial progenitor cells and reduces SCF.^140^ Furthermore, isolated MSCs from mice exposed to cigarette smoke have increased expression of the Notch ligand, Jagged-1.^137^ Balanced levels of Notch signaling appear necessary to avoid development of hematological malignancies.^141^

Heavy smokers have significantly higher plasma levels of C-reactive protein and IL-6 compared with nonsmokers suggesting a systemic inflammatory response in addition to the local inflammation in the airways. Inflammation, as described earlier, leads to drastic alterations in hematopoietic output, which may be the reason for the increased blood cell counts observed in smokers. Furthermore, cigarette smoke contains high concentrations of ROS^142^ and induces ROS and DNA damage in endothelial cells.^143^

Cigarette smoke contains more than 5000 chemical compounds^144^ of which 93 are listed as harmful or potentially harmful by the Food and Drug Administration.^145^ Some of these are nicotine, cadmium, lead, acetaldehyde, and benzene, which may all directly affect the HSCs and their niches.

Nicotine, the addictive compound of tobacco, has been reported to increase the number of human MSCs in vitro^140^ and the number of endothelial progenitors cells and SCF in plasma from mice.^146^ Whether nicotine exhibits a direct effect on HSCs is still unclear, but 1 study reports the expression of a nicotinic acetylcholine receptor on HSCs, and that oral nicotine administration in mice leads to higher frequency of HSCs in BM and increased leukocyte counts in peripheral blood, BM, and spleen.^136^ Furthermore, nicotine is extensively metabolized to a number of compounds with cotinine being the most predominant.^133^ Cotinine has a much slower clearance than nicotine but studies investigating the effect of cotinine on the BM are very limited.

Cigarette smoking is a major exposure route for cadmium and to a lesser extent lead.^147^ Both are heavy metals with long half-lives, considerable toxicity, and are known carcinogens.^148^ Epidemiological studies have shown that cadmium exposure causes bone damage and increases the risk of osteoporosis.^149,150^ Cadmium induces endothelial dysfunction,^151^ increases the number of BMAs,^152^ suppresses osteogenic differentiation of BM MSCs,^153^ and induces DNA damage in MSCs affecting cell viability^154^ in vitro, suggesting a toxic effect of cadmium on the HSC niches.

Bone is a major reservoir of lead and contains more than 90% of the total lead body burden.^155^ In birds, areas with trabecular bone and BM accumulate high levels of lead.^156^ Lead exposure is cytotoxic to mouse BM MSCs causing increased DNA damage, reduced proliferation^157^ and MSCs numbers.^158^ Furthermore, long-term low-dose lead exposure in mice results in reduced bone density and trabecular bone.^158^ Lead suppresses proliferation of HSCs^159^ and myeloid and lymphoid differentiation^160,161^ in mice. In contrast, human smokers have increased numbers of monocytes and granulocytes and comparable levels of lymphocytes to nonsmokers.^162^ The level of exposure to heavy metals in smoke drawn from a single cigarette is small and likely not acutely toxic, but the accumulation of these metals in the body over years of exposure is a health concern,^147^ and may cause harmful changes to the HSC niches.

Benzene is a known carcinogen and a serious public health concern. After inhalation, benzene metabolites are distributed to lipid-rich and well-perfused tissues including the BM. Multiple studies have linked benzene exposure to reduced leukocytes, erythrocytes, neutrophil, and lymphocytes counts.^163,164^ Benzene metabolites significantly impair survival of cultured human BM MSCs^165^ and alter the function of human BM endothelial cells.^166,167^ Furthermore, benzene increases the risk of a broad range of hematological malignancies and disorders.^168^

**Figure 3.**
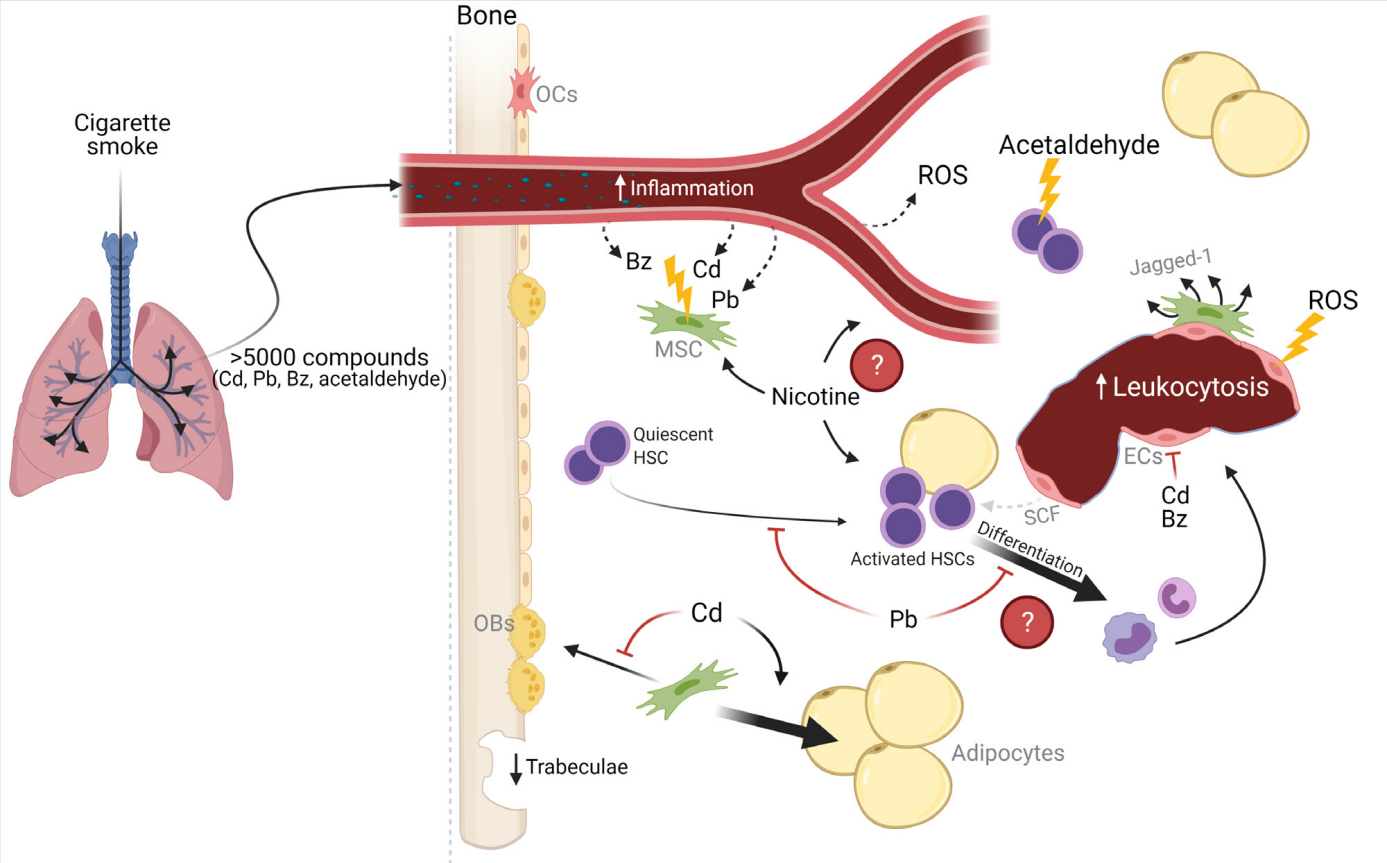
**Hypothetical working model for cigarette smoke–induced niche changes in human BM.** Inhaled tobacco smoke reaches the airways, and nicotine and many other compounds are absorbed into the bloodstream including Cd, Pb, Bz, and acetaldehyde. Smoking induces leukocytosis suggesting HSC activation. Furthermore, smoking increases systemic inflammation and ROS concentrations. Cigarette smoke damages the endothelial cells, possibly by ROS-induced DNA damage or Cd and/or Bz-toxicity and reduces the levels of SCF. Similarly, the survival of MSCs is impaired, possibly due to DNA damage induced by Cd, Pb, or Bz, and they show increased Jagged-1 expression. Acetaldehyde is directly hematotoxic and induces DNA damage in HSCs. Pb suppress proliferation and differentiation of HSCs. However, this is contradictory to the increased leukocytosis observed in smokers. Long-term exposure of Pb reduces trabecular bone volume. Cd suppresses osteogenic and induces adipogenic differentiation of MSCs leading to an expansion of BMAT. BM = bone marrow; BMAT = bone marrow adipose tissue; Bz = benzene; Cd = cadmium; ECs = endothelial cells; HSC = hematopoietic stem cell; MSC = mesenchymal stromal cell; OBs = osteoblasts; OCs = osteoclasts; Pb = lead; ROS = reactive oxygen species; SCF = stem cell factor. Created with BioRender.

In summary, cigarette smoking introduces several toxic and carcinogenic compounds to the body which may impair hematopoiesis through direct hematotoxicity and potentially by disrupting the HSC niches (Figure 3). However, some inconsistencies remain between the observed effect of the individual compounds of cigarette smoke on BM in animal models, and the clinical observations in heavy smokers. Thus, there is still a knowledge gap of how and if the many compounds in cigarette smoke reaches the BM and if so, how together they affect the HSC niche.

## Perspectives and conclusion

Several lifestyle factors, such as smoking and obesity, are associated with increased risk of developing a hematological cancer. However, there is currently limited knowledge of how lifestyle factors affect hematopoiesis. Especially the number of human studies is limited as this topic has received little attention.

Here we provide an overview of the current knowledge about the potential consequences of unhealthy diet, chronic alcohol consumption, and smoke exposure on the HSC niches and hematopoiesis. The data suggest that HFD, alcohol, and smoking cause inflammation, increase BMAT, and induce niche remodeling either through structural changes or changes in expression of niche factors.

Furthermore, we present data suggesting the existence of multiple HSC niches and a dynamic transition of HSCs between these niches dependent on the individual state of the HSC. Whether HFD, alcohol, or smoke influence the localization of HSCs to different niches and/or the ability of the niche to induce either quiescence or activation of the HSCs is a subject for further studies.

Most of the data presented here are from animal studies or based on correlations observed in humans. Therefore, any mechanistic relationship between unhealthy lifestyle and malignant transformation remains hypothetical and an interesting target for future research. This could potentially reveal new pathogenic mechanisms, but also identify novel therapeutic or prophylactic targets and approaches.

In conclusion, HSCs ability to sustain a normal hematopoiesis is highly dependent of the support from the HSC niches. Unhealthy lifestyle such as HFD, excessive alcohol intake, and smoking induce alterations in the BM. However, more studies, especially in humans, are needed to fully understand the impact of lifestyle on the HSC niches and how this may be linked to malignant transformation.

## Disclosures

The authors have no conflicts of interest to disclose.
